# The Prognostic Value of CIP2A and Its Association with CD31, E-Cadherin, and pAMPK in Lung Cancer

**DOI:** 10.3390/ijms26178362

**Published:** 2025-08-28

**Authors:** Peng Yu Lee, Ching-Yu Shih, Chiao-Yin Cheng, Hua Ho, Yen-Lin Chen, Chih-Jung Chang

**Affiliations:** 1 Department of Emergency Medicine, Far Eastern Memorial Hospital, New Taipei 220, Taiwan; femh97681@femh.org.tw (P.Y.L.); femhr9969@femh.org.tw (C.-Y.C.); femh97657@femh.org.tw (H.H.); 2 Center for Precision Medicine and Genomics, Tri-Service General Hospital, National Defense Medical University, Taipei 114, Taiwan; c588011124@tsghms.ndmctsgh.edu.tw; 3 Department of Pathology, Center for Precision Medicine and Genomics, Tri-Service General Hospital, National Defense Medical University, Taipei 114, Taiwan; 4 Graduate Institute of Medicine, Yuan Ze University, Taoyuan City 320, Taiwan

**Keywords:** CIP2A, lung cancer, CD31, AMPK, AKT

## Abstract

Cancerous inhibitor of protein phosphatase 2A (CIP2A) is an oncoprotein promoting tumor progression via multiple pathways. Its prognostic significance in lung cancer remains unclear. We analyzed tumor samples from 53 patients with lung cancer undergoing curative surgical resection without prior chemotherapy or radiotherapy. Immunohistochemical staining and H-score quantification were performed to assess CIP2A and related protein expression. Patients were stratified based on CIP2A expression (cutoff value = 218.33). Kaplan–Meier survival analysis produced curves and log-rank tests. Correlations with clinicopathological and molecular markers were assessed. High CIP2A expression was significantly associated with poorer survival (log-rank, *p* = 0.0051). Pearson correlation analysis revealed that CIP2A expression was positively correlated with clusters of differentiation 31 (r = 0.420, *p* = 0.002), epithelial cadherin (r = 0.372, *p* = 0.006), and phosphorylated protein kinase B (r = 0.332, *p* = 0.015), and negatively correlated with phosphorylated AMP-activated protein kinase (r = −0.474, *p* < 0.001), suggesting potential roles for CIP2A in promoting angiogenesis, sustaining epithelial traits, and suppressing metabolic regulation via AMPK signaling. CIP2A is a significant prognostic biomarker in lung cancer, contributing to tumor progression through modulation of angiogenesis and metabolic pathways. Exploration of its therapeutic potential and underlying mechanisms is warranted.

## 1. Introduction

Lung cancer is one of the most common and deadliest malignancies worldwide. In 2020, it accounted for over 2.2 million new cases and 1.8 million deaths globally, representing approximately 18% of all cancer-related deaths [1,2]. Although countries like the United States have seen declines in lung cancer mortality due to anti-smoking campaigns and early screening, the global burden remains substantial, particularly in Asia [3]. Major risk factors for lung cancer include smoking, second-hand smoke, air pollution (e.g., PM2.5), radon exposure, and asbestos. In East Asia, despite declining smoking rates, the incidence of lung adenocarcinoma among never-smokers—especially women—continues to rise, highlighting the critical role of environmental and genetic factors [4,5,6]. In Taiwan, lung cancer remains a leading cause of cancer death. The annual incidence among women continues to increase, with a high proportion of cases in never-smokers—over 60%—and adenocarcinoma being the predominant histologic type, accounting for approximately 50% of cases in men and up to 90% in women [7,8]. Environmental exposures such as air pollution, indoor cooking fumes, occupational hazards, and genetic predisposition are key contributors [9]. In response, the Taiwanese government has implemented the Taiwan Lung Cancer Screening in Never Smoker Trial (TALENT) program, which uses low-dose computed tomography (LDCT) to screen high-risk individuals. The first-round screening identified a 2.1% lung cancer detection rate, most of which were early-stage cases, demonstrating the potential of LDCT to improve outcomes [10]. With the expansion of screening and the introduction of targeted and immunotherapies, the 5-year survival rate for lung cancer in Taiwan improved from 24% (2011–2015) to 40% (2017–2021), though it still lags behind that of other high-income countries [11].

The development of lung cancer is closely linked to a variety of genetic factors. Genome-wide association studies have identified multiple susceptibility loci, especially among East Asian never-smokers with lung adenocarcinoma. One study revealed 12 novel risk variants, bringing the total to 28 across 25 loci [12]. These findings support the use of polygenic risk scores for risk stratification and targeted screening [13].

Multiple protein biomarkers have been identified with prognostic value in lung cancer. Tissue inhibitor of metalloproteinases 1 (*TIMP1*) serves as an early detection and prognosis biomarker for aggressive lung cancer—its serum level correlates with tumor burden and survival in mice, and has been validated in clinical samples [14]. The membrane protein tight junction protein 1 is highly expressed in lung cancer, and its knockdown significantly reduces cell invasion, migration, and proliferation, suggesting its potential as a prognostic and therapeutic target [15]. Moreover, elevated *PD-L2* expression predicts poorer overall survival post-surgery, especially in patients with lung adenocarcinoma [16]. In stage I lung adenocarcinoma, a five-protein signature stratifies patients into high-risk groups with hazard ratios of 3.5–4.6, supporting its use in adjuvant treatment planning [17]. Chronic inflammation markers, such as C-reactive protein and its binding proteins, have also been linked to lung cancer prognosis [18]. Furthermore, elevated soluble programmed death-1 levels in plasma following immunotherapy correlate with improved overall survival, indicating its promise as a prognostic biomarker for immune checkpoint treatment [19]. Together, these protein-based studies lay the groundwork for personalized prognostic stratification and therapeutic decision-making in lung cancer.

Cancerous inhibitor of protein phosphatase 2A (PP2A) (CIP2A) is an endogenous inhibitor of PP2A that is highly overexpressed across most solid and hematologic tumors, correlating with poor prognosis [20,21]. It promotes oncogenesis primarily by inhibiting PP2A-mediated dephosphorylation of c-Myc, thereby stabilizing c-Myc to enhance proliferation, invasion, migration, and drug resistance [20,21]. A recent study in NSCLC further revealed that CIP2A binds to pyruvate kinase M2 (PKM2), inducing tetramer formation, redirecting PKM2 to mitochondria, and enhancing oxidative phosphorylation and B-cell lymphoma 2 (Bcl-2) phosphorylation—thereby boosting tumor cell energy metabolism and survival [22]. Additionally, a pan-cancer integrative analysis identified several regulatory single-nucleotide polymorphisms (e.g., rs1239349555, rs1576326380, and rs56255137) in the 5′/3′ untranslated regions (UTRs) of *KIAA1524* that are associated with CIP2A overexpression, and linked CIP2A regulatory networks with tumor protein p53 (*TP53*) mutations [23]. These novel findings position CIP2A not only as a robust prognostic biomarker but also as a promising therapeutic target, especially for interventions targeting metabolic vulnerabilities and personalized oncology strategies.

Despite these findings, no prior studies have systematically evaluated CIP2A as a prognostic biomarker or incorporated it into a predictive model for lung cancer outcomes. Therefore, the aim of our study was to investigate the prognostic value of CIP2A in lung cancer and to develop a prognostic model based on its expression. This may provide a novel tool for clinical risk stratification and personalized treatment planning.

## 2. Results

**Figure 1:**
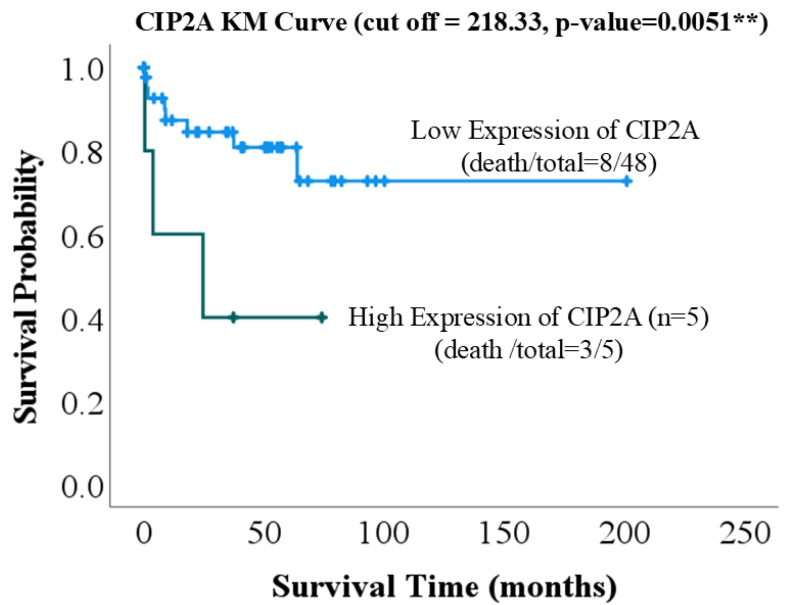
Kaplan–Meier survival curve stratified by CIP2A expression levels. The blue line represents high CIP2A expression, whereas the green line represents low CIP2A expression. CIP2A: cancerous inhibitor of PP2A; KM: Kaplan–Meier. ** *p*-value < 0.01.

This Kaplan–Meier survival curve illustrates the overall survival probabilities of patients with lung cancer stratified by CIP2A expression levels, using a cutoff point of 218.33. The median CIP2A level was 174.95 (Q1: 149.5, Q3: 202.0). In the low-expression group, the median CIP2A level was 173.50 (Q1: 143.2, Q3: 193.0), whereas in the high-expression group, the median CIP2A level was 222.0 (Q1: 220.8, Q3: 226.9). Patients with high CIP2A expression (blue line) demonstrated markedly lower survival probabilities compared to those with low CIP2A expression (orange line). The shaded areas represent 95% confidence intervals. The separation between the two curves was statistically significant, as determined by the log-rank test (*p* = 0.0051), indicating that high CIP2A expression is significantly associated with poorer prognosis (Figure 1). These results suggest that the level of CIP2A expression has a significant impact on patient survival. In the following sections, we explore potential biological mechanisms that may explain this observation.

To investigate the clinical significance of CIP2A expression in lung cancer, we first analyzed its association with various clinicopathological characteristics (Table 1). In terms of sex distribution, 62.5% of patients in the low CIP2A expression group were female and 37.5% were male. In contrast, the high expression group included 60.0% female and 40.0% male patients, with no statistically significant difference (*p* = 0.913). Regarding tumor grading, the high CIP2A expression group was predominantly composed of moderately differentiated tumors (80.0%) and contained no well-differentiated cases. In contrast, the low expression group consisted of 10.4% well-differentiated, 60.4% moderately differentiated, and 29.2% poorly differentiated tumors; however, the difference was not statistically significant (*p* = 0.627). For cancer staging, all patients with high CIP2A expression were in early stages (I or II), compared to 77.1% in the low expression group, with the remaining 22.9% distributed across stages III and IV (*p* = 0.485). These findings suggest no significant associations between CIP2A expression and sex, tumor differentiation, or stage, possibly due to the small sample size, especially within the high expression group (n = 5).

**Figure 2:**
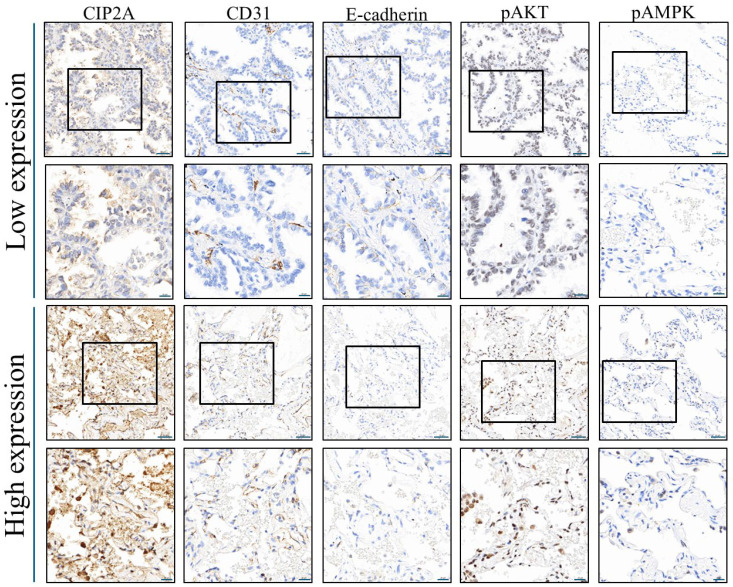
Immunohistochemical staining of the following markers in lung cancer tissues exhibiting high and low CIP2A expression: CD31, E-cad, p-Akt, and pAMPK. The boxed areas indicate locally magnified images. The first and third rows are shown at 200× magnification with a scale bar of 50 µm, while the second and fourth rows are shown at 400× magnification with a scale bar of 20 µm. CIP2A: cancerous inhibitor of protein phosphatase 2A; CD31: cluster of differentiation 31; E-cad: epithelial cadherin; p-Akt: phosphorylated protein kinase B; pAMPK: phosphorylated AMP-activated protein kinase.

We further compared the expression of molecular markers between the two groups to identify potential biological correlates of CIP2A expression (Table 2 and Figure 2). Although caspase-3 and Ki67 levels were comparable between the groups (*p* = 1.000 and 0.192, respectively), a significantly higher cluster of differentiation 31 (CD31) expression was observed in the high CIP2A group [111.0 (43.0–153.6)] compared to the low expression group [23.2 (11.4–45.6); *p* = 0.004], suggesting a role in promoting angiogenesis. Epithelial cadherin (E-cadherin) also trended higher (*p* = 0.060), whereas neural cadherin (N-cadherin) showed a borderline decrease (*p* = 0.069), hinting at possible involvement in epithelial–mesenchymal transition regulation. Most other markers, including fibronectin, phosphorylated protein kinase B (pAkt), phosphorylated extracellular signal-regulated kinase (pErk), and phosphorylated signal transducer and activator of transcription 3 (pStat3), showed no significant differences. However, phosphorylated AMP-activated protein kinase (pAMPK) expression was significantly lower in the high CIP2A group [3.8 (3.5–7.3)] than in the low group [8.3 (5.7–11.0); *p* = 0.018], suggesting a potential link between CIP2A and suppressed AMPK pathway activity.

To further evaluate potential predictors of high CIP2A expression, we conducted univariable logistic regression analysis (Table 3). None of the demographic, pathological, or molecular markers demonstrated statistically significant associations with high CIP2A expression (all *p*-values > 0.05). For example, male sex had an odds ratio (OR) of 1.24 (*p* = 0.750), age had an OR of 0.99 (*p* = 0.792), and molecular markers such as CD31 (OR = 0.99, *p* = 0.274) and pAMPK (OR = 0.99, *p* = 0.870) showed no significant predictive value. Due to the absence of statistically significant findings in univariable models, multivariable logistic regression was not performed.

Given the limitations of the logistic regression analysis, we performed Pearson correlation analysis to explore potential linear relationships between CIP2A expression and biomarker levels (Table 4), which aligned with the group comparisons in Table 2. CD31 (r = 0.420, *p* = 0.002) and E-cadherin (r = 0.372, *p* = 0.006) showed significant positive correlations with CIP2A expression, supporting a role in angiogenesis and epithelial characteristics. Additionally, pAkt expression was positively correlated with CIP2A (r = 0.332, *p* = 0.015), suggesting possible activation of oncogenic signaling. Conversely, pAMPK expression was negatively correlated with CIP2A (r = –0.474, *p* < 0.001), consistent with the earlier finding that high CIP2A expression is associated with suppressed AMPK activity. Other markers, such as caspase-3, Ki67, N-cadherin, fibronectin, pErk, and pStat3, showed no significant correlation.

Together, these results suggest that high CIP2A expression in lung cancer may be associated with enhanced angiogenesis (CD31), increased epithelial markers (E-cadherin), and suppression of AMPK-mediated metabolic regulation, providing mechanistic insights into its potential role in tumor progression.

## 3. Discussion

Our study demonstrates that high CIP2A expression is significantly associated with poorer overall survival in patients with lung cancer, as shown by Kaplan–Meier survival analysis. To identify potential factors influencing CIP2A expression, we initially performed logistic regression analysis across a panel of candidate proteins; however, none reached statistical significance. Consequently, we conducted Pearson correlation analysis, which revealed significant associations between CIP2A expression and three proteins: CD31, E-cadherin, and pAMPK. In the following sections, we explore potential biological connections between these markers, CIP2A, and lung cancer pathogenesis.

Specifically, high CIP2A expression correlated with enhanced angiogenesis (elevated CD31), increased epithelial marker expression (E-cadherin), and suppressed metabolic regulation (reduced pAMPK). These findings align with previous reports that CIP2A supports tumor metabolism by interacting with PKM2 to promote tetramer formation and mitochondrial localization, thereby enhancing oxidative phosphorylation and Bcl-2 phosphorylation to inhibit apoptosis [22]. Chen et al. also demonstrated that CIP2A enhances arginine biosynthesis and promotes metastasis in NSCLC, partly via mutant TP53 [24]. To our knowledge, no prior studies have directly linked CIP2A with CD31; thus, our results are the first to suggest a role for CIP2A in promoting angiogenesis, offering novel insights into its contribution to lung cancer progression.

Multiple studies have highlighted CIP2A’s role in NSCLC progression and treatment resistance. CIP2A promotes PKM2 tetramer formation, enhances oxidative phosphorylation, and drives metabolic reprogramming [22]. Its overexpression is associated with chemoresistance, whereas CIP2A silencing sensitizes NSCLC cells to cisplatin, suppresses proliferation and colony formation, and promotes apoptosis, accompanied by reduced Akt activation [25]. CIP2A is also a key mediator of erlotinib-induced apoptosis in EGFR–wild-type NSCLC [26], and modulation of CIP2A by bortezomib can overcome erlotinib resistance [27]. These findings emphasize CIP2A’s significance in metabolic regulation, chemotherapy sensitivity, and targeted therapy resistance, supporting its potential as a prognostic biomarker and therapeutic target.

CD31 (PECAM-1) is a well-established endothelial cell marker used to assess microvessel density and tumor angiogenesis. In NSCLC, CD31 expression correlates with tumor progression and prognosis. For example, CD31 positively associates with VEGF-A expression, supporting its role in tumor-induced neovascularization [28]. Co-expression of CD31 and nucleolin (CD31^high/NCL^high) predicts significantly worse disease-free survival in early-stage NSCLC [29], and dual immunostaining of CD31 with α-SMA can stratify vascular maturity and patient risk [30]. In our study, CIP2A expression correlated positively with CD31 levels (*p* = 0.004), suggesting a potential link between CIP2A and angiogenic activity in the lung tumor microenvironment. While the molecular mechanisms remain unclear, this observation warrants further investigation into whether CIP2A influences angiogenesis through downstream signaling or metabolic regulation.

Although E-cadherin is classically regarded as a tumor suppressor and EMT inhibitor—typically downregulated during tumor progression [31,32]—emerging evidence indicates context-dependent roles. Tumor cells may retain high E-cadherin to facilitate extracellular matrix or endothelial interactions, promoting collective migration rather than single-cell EMT [33]. In advanced EGFR-mutant lung adenocarcinoma, high E-cadherin expression has been paradoxically linked to worse prognosis and increased brain metastasis [34], suggesting pro-survival or pro-metastatic functions under certain molecular contexts. Our finding of a positive correlation between CIP2A and E-cadherin may reflect an intermediate epithelial–mesenchymal phenotype, in which epithelial traits aid survival, adhesion, and vascular interaction. Given CIP2A’s activation of oncogenic and metabolic pathways (e.g., c-Myc, AKT, PKM2), E-cadherin expression in this setting may represent phenotypic adaptation rather than tumor suppression.

Our results also support previous evidence that CIP2A sustains AKT signaling through PP2A inhibition. CIP2A prevents PP2A-mediated dephosphorylation of AKT at Ser473, maintaining p-AKT levels and promoting downstream signaling via GRP78 and mTOR. Lei et al. demonstrated that CIP2A overexpression enhances EGF-induced AKT phosphorylation in NSCLC models [35], while CIP2A knockdown reduces p-AKT and downstream oncogenic activity [36,37]. Similar mechanisms have been reported in multiple myeloma and colorectal cancer, where CIP2A silencing decreases p-AKT and increases drug sensitivity [38,39,40]. Consistent with these reports, we found a significant positive correlation between CIP2A and p-AKT expression, reinforcing the clinical relevance of this pathway. Moreover, high CIP2A expression was associated with decreased pAMPK levels, consistent with the suppressive effect of AKT–mTOR signaling on AMPK activity [41].

Based on current evidence, CIP2A does not directly suppress AMPK activation. In hepatocellular carcinoma cells, AMPK acts upstream to drive the sAC–cAMP–PKA–CREB/ATF1 cascade, as shown in aspirin-treated models where AMPK activation increases cAMP and PKA activity, and AMPK knockdown abolishes this effect [42]. In contrast, CIP2A functions downstream by inhibiting PP2A, thereby modulating CREB/ATF1 dephosphorylation without affecting PKA activity, as demonstrated in berbamine-treated cells [42]. In other cancers, such as prostate cancer, CIP2A predominantly exerts oncogenic effects through the PP2A–ERK/Akt/c-Myc axis, with no evidence of AMPK regulation [20]. Thus, CIP2A likely influences AMPK-related pathways indirectly via PP2A targets. The observed associations between CIP2A and increased E-cadherin and CD31 suggest that sustained AKT signaling may also affect epithelial phenotype and angiogenesis.

Pharmacologic or genetic inhibition of CIP2A in NSCLC relieves PP2A suppression, reduces AKT phosphorylation, and attenuates malignant phenotypes. Cucurbitacin B degrades EGFR and downregulates the CIP2A/PP2A/AKT axis, inhibiting growth and invasion of gefitinib-resistant NSCLC and reducing xenograft tumor volume [43]. Oridonin suppresses the CIP2A/AKT and EGFR/ERK pathways in gefitinib-resistant lung cancer [44]. TD-19 and afatinib induce apoptosis and tumor suppression in EGFR wild-type or mutation-negative NSCLC through CIP2A inhibition, with afatinib also blocking ELK-1-mediated CIP2A transcription [45,46]. Polyphyllin I/VII inhibits proliferation, migration, and EMT in A549/DDP-resistant cells, upregulates E-cadherin, and restores cisplatin sensitivity [26,47]. MicroRNA-383-5p directly targets CIP2A to suppress lung adenocarcinoma proliferation [48]. Although direct evidence linking CIP2A modulation to changes in CD31 and p-AMPK in lung cancer is limited, the cumulative data on AKT activation, E-cadherin regulation, and tumor progression support the hypothesis that CIP2A promotes angiogenesis and suppresses metabolic regulation. We aim to validate this by manipulating CIP2A in A549, H1299, and H460 cells, assessing CD31, E-cadherin, p-AKT, p-AMPK, and VEGFA, and performing endothelial tube formation assays, Seahorse metabolic analysis, and pharmacological rescue experiments.

Collectively, our data support a model in which CIP2A promotes lung cancer progression via coordinated regulation of p-AKT, AMPK suppression, and tumor microenvironment remodeling. Further mechanistic studies in vitro and in vivo are needed to elucidate how CIP2A integrates these signaling axes. Our observed inverse correlation between CIP2A and pAMPK aligns with emerging insights that CIP2A can disrupt PP2A-mediated AMPK signaling. CIP2A impedes specific AMPK-dependent phosphorylation events downstream of PP2A, such as ULK1 Ser555, leading to partial decoupling of AMPK-driven autophagy [49]. Compounds such as cucurbitacin B [41,50] and berbamine [42] reduce CIP2A expression, reactivate PP2A, inhibit mTORC1, and restore AMPK activity to promote autophagy and apoptosis. These findings support a model in which CIP2A overexpression drives metabolic reprogramming in lung cancer through dual modulation of the AKT–AMPK axis, consistent with our observation of reduced p-AMPK and elevated p-AKT in CIP2A-high tumors.

In NSCLC with high CIP2A and suppressed pAMPK, the downstream effects largely reflect the removal of AMPK’s metabolic “brakes”: (i) disinhibition of mTORC1, enhancing protein synthesis and growth signaling [49]; (ii) increased de novo lipogenesis via reduced ACC phosphorylation, supporting membrane biogenesis and proliferation [49]; (iii) impaired ULK1-driven autophagy/mitophagy, altering stress tolerance and therapy response [49]; (iv) a metabolic shift toward anabolic programs (glycolysis and biosynthesis) that favor tumor progression [30,51]; (v) oncogenic crosstalk (e.g., MAPK/ERK) that AMPK normally restrains, promoting proliferation, EMT, and invasiveness [51,52]; and (vi) modulation of the tumor microenvironment and immunity, where loss of AMPK signaling is associated with pro-tumor metabolic reprogramming and immunosuppressive niches [53,54]. Overall, pAMPK inhibition downstream of high CIP2A may enhance anabolic growth, invasiveness, and therapy resistance in NSCLC, while recognizing AMPK’s context-dependent roles [30,49,51].

In summary, our findings underscore CIP2A’s multifaceted role in lung cancer progression. By regulating p-AKT, pAMPK, CD31, and E-cadherin, CIP2A integrates oncogenic signaling, metabolic suppression, angiogenesis, and epithelial plasticity. These results reinforce CIP2A as a driver of poor prognosis and a central hub linking proliferative, metabolic, and microenvironmental cues in lung tumorigenesis. Further mechanistic dissection of these interactions may reveal novel therapeutic strategies targeting the CIP2A axis.

## 4. Materials and Methods

We collected tumor specimens from 53 patients with lung cancer who underwent their first surgical resection between 2000 and 2014. None of the patients had received chemotherapy, radiotherapy, or targeted therapy before surgery. The study protocol adhered to the principles of the Declaration of Helsinki and was approved by the Institutional Review Board of Cardinal Tien Hospital on 27 June 2018, as a study exempt from informed consent (Approval No. CTH-106-5-042).

From each of the 53 lung cancer samples, a cylindrical core measuring 20 mm in diameter was extracted, rearranged, and embedded into a tissue microarray block. Sections were then cut at a thickness of 5 μm and mounted onto saline-coated glass slides, which were stored in a cool, dry place for later use. Before immunohistochemical staining, the slides were heated at 65 °C for 1 h to melt the paraffin, followed by deparaffinization. The deparaffinization process involved sequential immersion in xylene for 10 min (twice), followed by 100% ethanol for 5 min, 95% ethanol for 5 min, and 75% ethanol for 5 min, followed by a 10 min rinse in running water.

The slides were then placed into an automated immunohistochemistry stainer (BenchMark XT automated stainer; Ventana, Tucson, AZ, USA). After initial rinsing with buffer solution, antigen retrieval was performed according to the specific antibody conditions for 24–48 min using a heated (90 °C) ethylenediaminetetraacetic acid buffer. After antigen retrieval, the slides were rinsed several times with buffer, followed by incubation with the primary antibody for 60 min at 37 °C. The primary antibodies and staining conditions were based on our team’s previous studies [55]. Subsequently, a secondary antibody was applied and incubated for 24 min. The signal was visualized using a 3,3′-diaminobenzidine chromogen substrate. Finally, the slides were air-dried to complete the staining process.

**Figure 3:**
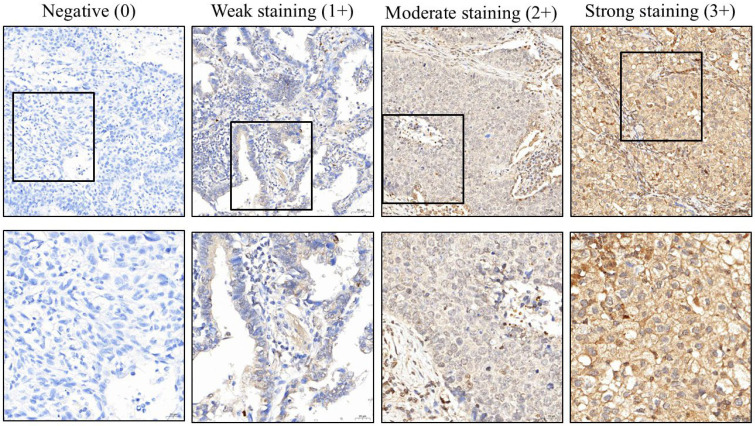
Immunohistochemical analysis of CIP2A expression in lung cancer tissues, illustrating varying levels of protein presence. CIP2A: cancerous inhibitor of PP2A. The black box shows a magnified view.

The stained slides were scanned using a slide scanner (3DHISTECH, Budapest, Hungary) at 200× magnification, and the proportion of stained tissue was quantified using computer counting software (CellQuant and PatternQuant, vertion 2.3; 3DHISTECH, Budapest, Hungary) as well as ImageJ software (version 1.50d, 25 October 2015; developed by the National Institutes of Health and the Laboratory for Optical and Computational Instrumentation, University of Wisconsin). Staining intensity was graded on a scale from 0 to 3, where 0 indicated a negative reaction and 3 represented the strongest staining intensity (Figure 3). The H-score was calculated by multiplying the staining intensity by the percentage of positive staining, and this score was used for subsequent analysis. Dr. Yen-Lin Chen reviewed the analysis outputs to check for any apparent abnormalities in the results, excluding any such cases, when necessary, but did not intervene in the scoring process. We performed Kaplan–Meier survival analysis for CIP2A using Python version 3.13.5 (released on 11 June 2025), incorporating the lifelines module. The optimal cutoff value identified was 218.33, and corresponding Kaplan–Meier survival curves and log-rank tests were generated. Subsequently, we used SPSS version 26.0 (IBM Corp., Chicago, IL, USA) for further statistical analyses. We first conducted preliminary checks on continuous variables to assess normality. Based on the results, only age followed a normal distribution; thus, it was expressed as mean ± standard deviation and analyzed using an independent samples t-test. All other continuous variables did not meet normality assumptions; therefore, they were presented as interquartile ranges and analyzed using the non-parametric Mann–Whitney U test. In addition, we also conducted univariable logistic regression analyses and Pearson correlation analyses. A *p*-value of less than 0.05 was considered statistically significant.

## 5. Conclusions

Our study demonstrates that high CIP2A expression is significantly associated with poorer overall survival in patients with lung cancer (log-rank *p* = 0.0051). CIP2A correlates positively with p-AKT, CD31, and E-cadherin, and negatively with pAMPK, suggesting its role in tumor proliferation, angiogenesis, epithelial plasticity, and metabolic suppression. Notably, this is the first study to link CIP2A with CD31, indicating a novel role in tumor-associated angiogenesis. These findings highlight CIP2A as a central regulator of oncogenic signaling and the tumor microenvironment, supporting its potential as a prognostic biomarker and therapeutic target.

## Figures and Tables

**Table 1:** Association between CIP2A expression levels and clinicopathological characteristics.

	Low Expression of CIP2A	High Expression of CIP2A	Total	*p*-Value
Sex				0.913
Female	30 (62.5%)	3 (60.0%)	33 (62.3%)	
Male	18 (37.5%)	2 (40.0%)	20 (37.7%)	
Age				
Grading				0.627
Well	5 (10.4%)	0 (0.0%)	5 (9.4%)	
Moderate	29 (60.4%)	4 (80.0%)	33 (62.3%)	
Low	14 (29.2%)	1 (20.0%)	15 (28.3%)	
Size				
Stage				0.485
I and II	37 (77.1%)	5 (100.0%)	42 (79.2%)	
IIIa	10 (20.8%)	0 (0.0%)	10 (18.9%)	
IIIb and IV	1 (2.1%)	0 (0.0%)	1 (1.9%)	

**Table 2:** Comparison of biomarker expression between low and high CIP2A expression groups.

	Low Expression of CIP2A	High Expression of CIP2A	Total	*p*-Value
Caspase3	4.6 (3.8, 7.9)	4.5 (3.4, 14.4)	4.5 (3.8, 7.9)	1.000
Ki67	3.8 (1.1, 13.7)	2.5 (0.2, 4.6)	3.3 (1.1, 12.7)	0.192
CD31	23.2 (11.4, 45.6)	111.0 (43.0, 153.6)	29.3 (11.9, 54.6)	0.004 **
E-cad	102.9 (100.7, 111.9)	110.8 (104.3, 134.6)	203.4 (101.1, 112.0)	0.060
N-cad	4.4 (2.7, 8.3)	2.7 (2.0, 3.9)	4.26 (2.6, 8.1)	0.069
Fibronectin	214.7 (158.3, 244.2)	186.4 (97.8, 239.6)	209.3 (154.2, 243.9)	0.432
pAkt	46.6 (17.2, 95.8)	116.4 (45.5, 159.6)	50.2 (17.5, 102.2)	0.181
pErk	3.5 (0.7, 22.0)	3.2 (1.3, 92.2)	3.2 (0.9, 21.6)	0.605
pStat3	1.6 (0.2, 11.5)	3.3 (1.9, 8.9)	1.9 (0.4, 11.1)	0.468
pAMPK	8.3 (5.7, 11.0)	3.8 (3.5, 7.3)	8.2 (5.1, 10.4)	0.018 *

* *p*-value < 0.05; ** *p*-value < 0.01.

**Table 3:** Univariable logistic regression analysis of factors associated with high CIP2A expression.

	Univariable	*p*-Value
Sex		
Female	Reference	
Male	1.24 (0.33–4.60)	0.750
Age	0.99 (0.94–1.05)	0.792
Grading		
Well	Reference	
Moderate	0.89 (0.08–9.44)	0.922
Low	2.00 (0.17–22.95)	0.578
Size	1.13 (0.84–1.51)	0.428
Stage		
I and II	Reference	
IIIa	0.31 (0.04–2.76)	0.296
IIIb and IV	0.00 (0.00–)	1.000
Marker		
Caspase3	1.03 (0.97–1.08)	0.346
Ki67	1.00 (0.99–1.02)	0.652
CD31	0.99 (0.97–1.01)	0.274
E-cad	1.00 (0.93–1.08)	0.997
N-cad	1.01 (0.98–1.04)	0.538
Fibronectin	1.00 (0.99–1.01)	0.748
pAkt	1.00 (0.99–1.01)	0.956
pErk	1.00 (0.99–1.01)	0.793
pStat3	0.96 (0.87–1.05)	0.356
pAMPK	0.99 (0.88–1.12)	0.870

**Table 4:** Analysis of the correlation coefficients between various biomarkers and high or low expression of CIP2A.

Marker	Pearson Correlation	*p*-Value
Caspase3	0.104	0.461
Ki67	−0.017	0.902
CD31	0.420	0.002 **
E-cad	0.372	0.006 **
N-cad	−0.118	0.400
Fibronectin	0.155	0.268
pAkt	0.332	0.015 *
pErk	0.165	0.236
pStat3	0.116	0.407
pAMPK	−0.474	<0.001 ***

* *p*-value < 0.05, ** *p*-value < 0.01, *** *p*-value < 0.001.

## Data Availability

The original data related to this study are available from the corresponding author upon reasonable request.

## Abbreviations

The following abbreviations are used in this manuscript:

Bcl-2	B-cell lymphoma 2
CD31	cluster of differentiation 31
CIP2A	cancerous inhibitor of PP2A
EGFR	epidermal growth factor receptor
E-cadherin	epithelial cadherin
LDCT	low-dose computed tomography
N-cadherin	neural cadherin
NSCLC	non-small-cell lung cancer
OR	odds ratio
pAkt	phosphorylated protein kinase B
pAMPK	phosphorylated AMP-activated protein kinase
pErk	phosphorylated extracellular signal-regulated kinase
pStat3	phosphorylated signal transducer and activator of transcription 3
PKM2	pyruvate kinase M2
PP2A	protein phosphatase 2A
TIMP1	tissue inhibitor of metalloproteinases 1
TP53	tumor protein p53
UTR	untranslated region
